# CRISPR/Cas9-Mediated Specific Integration of *Fat-1* and *IGF-1* at the p*Rosa26* Locus

**DOI:** 10.3390/genes12071027

**Published:** 2021-07-01

**Authors:** Wenni You, Mengjing Li, Yilin Qi, Yanbing Wang, Yiwu Chen, Ying Liu, Li Li, Hongsheng Ouyang, Daxin Pang

**Affiliations:** 1 Key Lab for Zoonoses Research, Ministry of Education, Jilin Provincial Key Laboratory of Animal Embryo Engineering, College of Animal Sciences, Jilin University, Changchun 130062, China; youwn18@mails.jlu.edu.cn (W.Y.); mjli18@mails.jlu.edu.cn (M.L.); qiyl9918@mails.jlu.edu.cn (Y.Q.); wangyb19@mails.jlu.edu.cn (Y.W.); chenyw20@mails.jlu.edu.cn (Y.C.); ying19@mails.jlu.edu.cn (Y.L.); li_li99@mails.jlu.edu.cn (L.L.); Ouyh@jlu.edu.cn (H.O.); 2 Chongqing Research Institute, Jilin University, Chongqing 401123, China

**Keywords:** CRISPR/Cas9, *Fat-1*, *IGF-1*, knock-in, pig

## Abstract

Many researchers have focused on knock-in pigs for site-specific integration, but little attention has been given to genetically modified pigs with the targeted integration of multiple recombinant genes. To establish a multigene targeted knock-in editing system, we used the internal ribosome entry site (IRES) and self-cleaving 2A peptide technology to construct a plasmid coexpressing the fatty acid desaturase (*Fat-1*) and porcine insulin-like growth factor-1 (*IGF-1*) genes at equal levels. In this study, pigs were genetically modified with multiple genes that were precisely inserted into the p*Rosa26* locus by using the clustered regularly spaced short palindrome repeat sequence (CRISPR)/CRISPR-related 9 (Cas9) system and somatic cell nuclear transfer technology (SCNT) in combination. Single copies of the *Fat-1* and *IGF-1* genes were expressed satisfactorily in various tissues of F0-generation pigs. Importantly, gas chromatography analysis revealed a significantly increased n-3 polyunsaturated fatty acid (PUFA) level in these genetically modified pigs, which led to a significant decrease of the n-6 PUFA/n-3 PUFA ratio from 6.982 to 3.122 (*** *p* < 0.001). In conclusion, the establishment of an editing system for targeted double-gene knock-in in this study provides a reference for the precise integration of multiple foreign genes and lays a foundation for the development of new transgenic pig breeds with multiple excellent phenotypes.

## 1. Introduction

With the rapid development of biotechnology, genetic modification technology has gradually been applied to animal breeding. In the past few years, major achievements have been made in improving the traits of transgenic pigs via the CRISPR/Cas9-mediated knock-in of foreign genes [1,2,3,4,5], providing unprecedented potential for accelerating pig agricultural breeding. Researchers have developed knock-in pigs expressing the *pRSAD2* [6], *UCP1* [7] and *PBD-2* [8] genes. The disease resistance, lean meat rate, and growth rate of these pigs have been improved. Moreover, the human *FGF2*(*hFGF2*) gene was knocked into the β-casein gene locus of the bovine embryo genome to induce the expression of hFGF2 protein in the bovine mammary gland [9]. Genetically modified hornless cows were produced by the genomic integration of the *POLT* gene, thereby avoiding the pain caused by dehorning [10]. However, the use of a single recombinant gene transgenic animal is not sufficient for improving two or more traits simultaneously, as the preparation of transgenic animals is very costly, time-consuming and laborious. Therefore, methods for simultaneously inserting multiple foreign genes to improve multiple transgenic animal traits are urgently needed.

Research has shown that n-3 PUFAs have indispensable physiological and health functions in the body [11]. They have been shown to have special preventive effects on cancer [12], cardiovascular diseases [13,14] and neurological diseases [15] in a variety of animal models. According to report, long-term intake of excessive n-6 polyunsaturated fatty acids may increase the possibility of breast cancer [16]. Therefore, maintaining a reasonable n-6 PUFAs /n-3 PUFAs ratio is particularly vital for maintaining health. *Fat-1* gene was originally found in Caenorhabditis elegans, it can encode a fatty acid desaturase, and this desaturase can convert n-6 PUFAs into n-3 PUFAs, including linolenic acid (ALA), dihydrofuran (DHA) and propylene oxide (EPA). However, the efficiency of DHA and EPA synthesis in the human body is limited [17], and these components are mainly obtained from the diet.

The insulin growth factor (IGF) system is mainly composed of *IGF-1, IGF-2*, insulin receptor, and insulin-like growth factor binding protein, among others [18]. Proinsulin-like growth factor 1 (*IGF-1*), a basic single-chain polypeptide composed of 70 amino acids, is one of the most important factors in the IGF family [19,20]. Previous studies have indicated that *IGF-1* promotes growth in animals. Liu found that the growth and development of *IGF-1* knockout mice were slower and that the adult body weight and body length of these mice were only approximately 30–60% of those of normal control mice. The use of growth hormone did not ameliorate these effects. In contrast, the treatment of control mice with growth hormone significantly increased their body weight and body length, indicating that *IGF-1* mediates the growth and development of growth hormone [21]. Moreover, IGF-1 is a key factor regulating the growth and development of skeletal muscle, and the muscle-specific upregulation of *IGF-1* leads to muscle hypertrophy [22,23,24,25,26]. To our knowledge, there are no transgenic pigs with *IGF-1* gene knock-in.

To achieve double-gene knock-in to the same transgenic pig locus, the *Fat-1* and *IGF-1* genes were coexpressed by IRES and self-cleaving 2A peptide technology. In this manner, a system was constructed that allowed multiple genes to express in coordination [27,28] such that multiple genes were knocked in at precisely the same specific locus. The IRES and class 2A cleavage sequences are polycistronic elements that play vital roles in the eukaryotic ectopic expression of foreign genes. A previous study used 2A self-shearing technology to construct a vector coexpressing the *IGF-1* and *Fat-1* genes and to prepare coexpressing mice [29]. The site of foreign gene insertion is critical for the preparation of genetically modified animals. The *Rosa26* gene was first discovered in mouse embryonic stem cells, and as a friendly site for gene editing, has been widely used in various transgenic animal studies [3,30].

Genetically modified pigs are vital to the development of the agricultural economy. In this research, we used the CRISPR/Cas9 editing system to specifically insert the *Fat-1* and *IGF-1* genes into the *Rosa26* locus of porcine fetal fibroblasts (PFFs), established a double-trait gene editing system and prepared double-trait gene transgenic pigs. Our research provides a reference for further research on transgenic pig preparation and genetic breeding.

## 2. Materials and Methods

### 2.1. Vector Construction

pX330-sgRNA91, a Cas9/sgRNA vector containing p*Rosa26*-specific sgRNA, was previously constructed and stored in our laboratory. In order to efficient expression in mammals, we optimized the codons of the *Fat-1* gene from C. elegans (GenBank: NM_001028389) and the *IGF-1* gene from pigs (GenBank: XM_005664196). Then, we sent the sequences of the splice acceptor (SA), optimized *Fat-1* and *IGF-1*, IRES, self-cleaving 2A peptide and SV40 PolyA to GENEWIZ (Suzhou, China) to synthesize the foreign expression cassette. Our laboratory has optimized the length of the homology arm (HA) at the p*Rosa26* locus. When the donor vector contains 3’HA with a length of 1012 bp and a 5’HA with a length of 510 bp for the p*Rosa26* site, the knock-in efficiency is higher [3]. So we amplified the optimized HA by Polymerase Chain Reaction (PCR) and inserted them into PUC57 vector (Addgene ID 51306). At last, we integrated the synthesized fragment IRES-IGF1-2A-Fat1 between the 5’HA and 3’HA, and designated this donor vector as PUC57-IRES-IGF1-2A-Fat1-KI.

### 2.2. Electroporation and Limiting Dilution Screening of PFFs

We suspended approximately 30 μg donor PUC57-IRES-IGF1-2A-Fat1-KI plasmid and 30 μg pX330-sgRNA91 plasmid in 200 μL Opti-MEM buffer (Gibco, Grand Island, NY, USA), and electroporated into 3 × 106 PFF cells according to the specified BTX-ECM 2001 parameters. 48 h later, the cells were seeded in 10 cm dishes at an average density of 3000 cells per dish. After approximately 8 days, the selected clones were cultured in a 24-well plate. Upon reaching 80% confluence, 20% of the single-cell clones were digested by trypsin and lysed with 10 µL of NP40 buffer (0.6% proteinase K & 0.4% NP40) under the following conditions: 1 h at 56 °C and 10 min at 95 °C. The cell lysate was used as template of PCR for genotyping each single cell clone. Primer 1F (5′-AGGCTTCTGGGTGGTGGTGAC-3′) was designed for the inserted gene fragment, and primer 1R (5′-GCAACGTGGCAGGAAGCG-3′) was designed outside the 3’HA. The PCR conditions were as following: 98 °C for 2 min; 35 cycles of 98 °C for 30 s, 64 °C for 30 s, and 68 °C for 30 s. the last step is keeping at 68 °C 5 min.

PCR was performed using TIANSeq HiFi Amplification Mix (TIANGEN, Beijing, China). The suspected positive cell clones were sequenced, and total RNA was then extracted from the positive cell clones with the TRNzol-A+ Reagent (TIANGEN, Beijing, China) kit. One microgram of total RNA was reverse transcribed into cDNA by FastKing one-step RT-PCR kit (TIANGEN, Beijing, China). The primers 2F/2R were designed for amplification of the *Fat-1* gene, and the 3F/R primers were designed for the *IGF-1* gene. Their sequences are as follows: 2F, 5′-ACCACATCGACAAGGACCAC-3′ and 2R, 5′-TGATGACGCACTGCACTCTT-3′; 3F, 5′-TGCTTGCTCTCCTTCACCAG-3′ and 3R, 5′-TCCAGCCTCCTCAGATCACA-3′. Next, PCR was performed, with 1 μL of cDNA serving as the template. The PCR conditions for amplifying *Fat-1* and *IGF-1* were as follows: 98 °C for 2 min; 35 cycles of 98 °C for 30 s, 58 °C for 30 s and 68 °C for 15 s; and a hold at 68 °C for 5 min as the last step.

### 2.3. SCNT and Embryo Transfer

According to previous research [31], the PFFs that were positive for the site-specific insertion of *IGF-1* and *Fat-1* genes were used for somatic cell nuclear transfer (SCNT). The reconstructed embryo was activated and cultured in PZM3 medium for approximately 18 h before being injected into the fallopian tube. After 28 days, the pregnancy status of the recipient sow was detected by an ultrasound detector.

### 2.4. Generation of Transgenic Pigs and Genotype Analysis

We extracted DNA from the ear tips of the F0-generation piglets to determine whether *Fat-1* and *IGF-1* were inserted at the *Rosa26* site. The F0-generation piglet DNA was as the template for PCR amplification using the primers 1F/R, as described above. Total RNA was extracted from the ear tips of the piglets using the primers 2F/R and 3F/R to determine whether the *Fat-1* and *IGF-1* genes were transcribed into their corresponding mRNAs. The RT-PCR amplification conditions were the same as those previously mentioned.

### 2.5. Transcriptional Analysis of the IGF-1 and Fat-1 Genes in Transgenic Pigs Using Real-Time PCR

We extracted total RNA of tissues from spleen, heart, kidney, lung, liver and muscle of *Fat-1* and *IGF-1* knock-in piglets by Animal Tissue Total RNA Extraction Kit (TIANGEN, Beijing, China). 1ug of total RNA from each tissue was converted into cDNA by using the FastKing one-step RT-PCR kit (TIANGEN, Beijing, China). The cDNA from each tissue was used as template of real-time fluorescence quantitative PCR to quantify the expression of the *IGF-1* and *Fat-1* genes at the transcription level. Porcine *GAPDH* was selected as reference gene for real-time PCR, and the primers for *IGF-1*, *Fat-1* and *GAPDH* were as following: *Fat-1* forward strand, 5′-ACCACATCGACAAGGACCAC-3′ and reverse strand 5′-TGATGACGCACTGCACTCTT-3′; *IGF-1* forward strand 5′-TGCTTGCTCTCCTTCACCAG-3′ and reverse strand 5′-TCCAGCCTCCTCAGATCACA-3′; and *GAPDH* forward strand 5′-GCCATCACCATCTTCCAGG-3′ and reverse strand 5′-TCACGCCCATCACAAACAT-3′. The real-time PCR was performed on Bio-Rad IQ5 instrument.

### 2.6. Western Blot Analysis

We extracted total proteins from the longissimus dorsi and gastrocnemius tissues from positive piglets, and the protein content was detected by using the BCA Protein Concentration Determination Kit (BOSTER, Wuhan, China). Western blot was then performed as previously described [30]. The total proteins from muscle tissue were separated by 15% SDS-PAGE gel electrophoresis and then transferred to 0.22 µm polyvinylidene difluoride (PVDF) membranes. Next, the PVDF membrane were incubated with 5% blocking solution (1 g of nonfat dry milk dissolved in 20 mL of TBST buffer) for 90 min at 22 °C and then with the anti-rabbit IGF1 primary antibody (1:5000, Abcam, England) overnight at 4 °C while shaking. Then, the PVDF membrane were incubated with a goat anti-rabbit secondary antibody (Beyotime, 1:1000, China) for 2 h at 22 °C before being detected with an ultrasensitive chemiluminescence reagent kit (Beyotime, Shanghai, China) and Western blot imaging equipment. The membrane containing GAPDH protein was removed and detected after processing.

### 2.7. Fatty Acid Analysis

To detect the activity of the PUFA dehydrogenase encoded by the *Fat-1* gene, we ground the muscle tissue into powder in liquid nitrogen and extracted the fatty acids as reported previously [32,33]. Briefly, we homogenized powdered muscle tissue in a mixture of methanol, chloroform, and double distilled water. And about 15 min later, we added chloroform and centrifuged the sample to separate the mixture. Next, we separated the lower phase, dried it under nitrogen and resuspended it in boron trifluoride methanol, heated it at 90 °C for 30 min, and then extracted it with pentane and double distilled water. We vortexed the mixture and recovered the upper phase, dried and resuspended it. Finally, we injected the sample into a gas chromatograph equipped with an SP-2560 capillary column (Sigma, St. Louis, MO, USA) analyzer to analyze the fatty acid composition. The temperature of the chromatographic column was initially set to 140 °C and kept for about 5 min, then we increased the temperature to 220 °C and kept for about 40 min. We identified the peaks of the resolved fatty acids by comparison with Fatty Acid Standards (Supelco 37 Component FAME Mix and Methylall-cis-7,10,13,16,19-docosapentaenonate, Sigma, St. Louis, MO, USA) and calculated the percentage of various fatty acids by using the peak area normalization method.

### 2.8. Statistical Methods

The data of this study were analyzed by the *t*-test of GraphPad Prism 8 software, and *p* < 0.05 indicated statistical significance.

## 3. Results

### 3.1. Construction of the Fat-1 and IGF-1 Donor Vector and CRISPR/Cas9-Mediated Integration of Fat-1 and IGF-1 into PFFs

**Figure 1:**
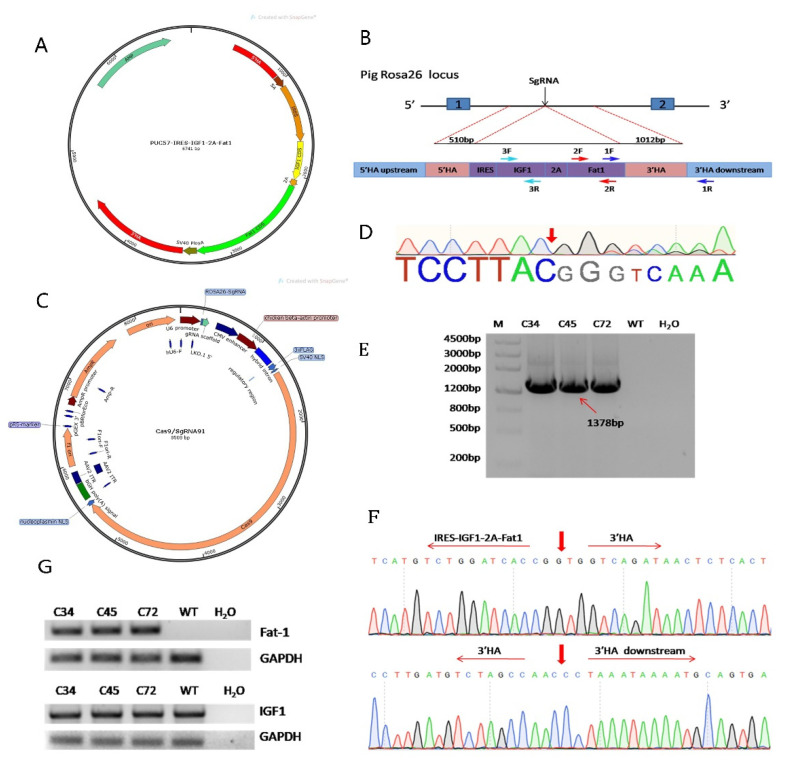
*IGF-1* and *Fat-1* were integrated into p*Rosa26* locus by homologous recombination. (**A**) Schematic diagram of IRES-*IGF1*-2A-*Fat1* inserted into the donor plasmid. (**B**) Strategy for the Cas9-mediated knock-in of *IGF-1* and *Fat-1* at p*Rosa26* site. (**C**) Schematic diagram of the Cas9/sgRNA plasmid. (**D**) The cleavage site of sgRNA at p*Rosa26* locus. (**E**) Screening of PFF clones by PCR with primer 1F/R. The primers 1F and 1R are designed in the upstream of 3’HA and downstream of 3’HA, respectively. M: DNA Marker III, WT: negative control. H_2_O: blank control. (**F**) Sanger sequencing of the products in (**E**). (**G**) RT-PCR was performed with primers 2F/R and 3F/R to confirm that the *IGF-1* and *Fat-1* genes were transcribed in the positive clones.

In our study, the *IGF-1* and *Fat-1* genes were cloned into the PUC57 vector, and IRES was added upstream of the *IGF-1* CDS. Similarly, the self-cleaving 2A peptide was added upstream of the *Fat-1* CDS and downstream of the *IGF-1* CDS (Figure 1A). To insert the *Fat-1* and *IGF-1* genes into the porcine *Rosa26* site (Figure 1B), the plasmids pX330-sgRNA91 (Figure 1C) and PUC57-IRES-IGF1-2A-Fat1-KI were co-transfected into PFFs by electroporation. The cleavage site of sgRNA91 is shown in Figure 1D. *Fat-1* and *IGF-1* knock-in PFFs were screened by the limiting dilution method. Briefly, 72 h after transfection, the cells were seeded in a 10 cm dish and cultured. After 9 days, the cultured single-cell clones were collected, and the knock-in event was confirmed by PCR with primers (1F/1R) located at both ends of the homology arm and Sanger sequencing. After HA-PCR, a total of 3 out of 85 single-cell clones showed the expected band. The sequencing results confirmed that single-cell clones 34, 45, and 72 were positive cell clones that exhibited *Fat-1* and *IGF-1* genes integration at the porcine *Rosa26* locus (Figure 1E,F). Finally, we extracted total RNA from these knock-in clones, and the results of RT-PCR proved that both the *Fat-1* and *IGF-1* genes were successfully transcribed and driven by the *Rosa26* promoter (Figure 1G).

### 3.2. F0 Generation of Knock-in Pigs and Transcriptional Analysis of the Fat-1 and IGF-1 Genes

**Figure 2:**
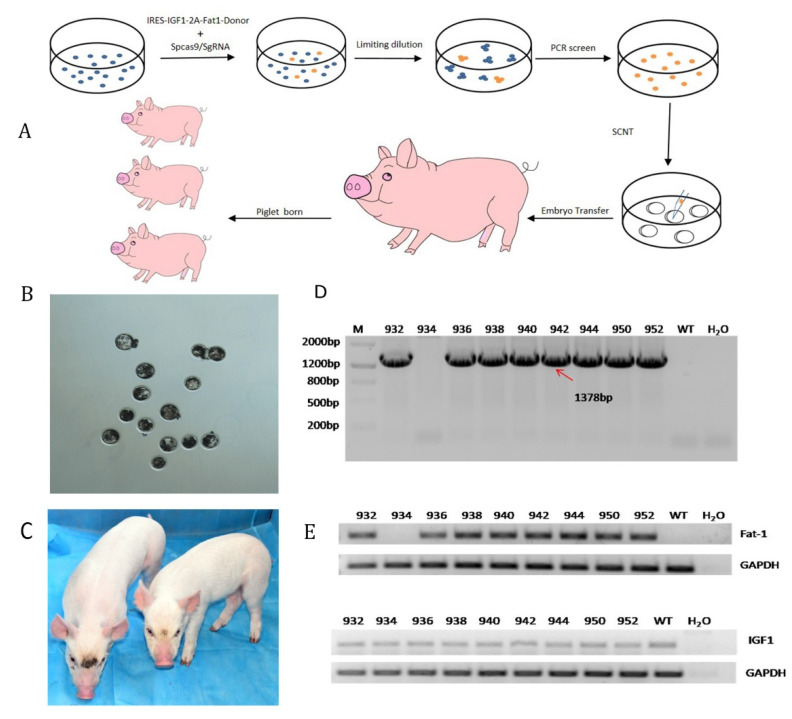
Generation and genotype identification of F0 transgenic piglets. (**A**) Flow chart of the experimental procedures for preparing *IGF-1* and *Fat-1* knock-in pigs. (**B**) To assess blastocyst development in vitro, the reconstructed embryos were cultured to the blastocyst stage in vitro. (**C**) Photo of F0 three-day-old piglets with site-specific *IGF-1* and *Fat-1* gene knock-in. (**D**) PCR analysis of *IGF-1* and *Fat-1* knock-in pigs using the primers 1F/1R (mentioned above): M: DNA 2000, WT: negative control, H_2_O: blank control. (**E**) RT-PCR analysis of *IGF-1* and *Fat-1* positive pigs using the primers 2F/R and 3F/R, respectively.

The fetal fibroblasts exhibiting *IGF-1* and *Fat-1* knock-in at the porcine *Rosa26* locus were further used for SCNT to produce transgenic pigs with dual traits (Figure 2A). Before SCNT, knock-in and wild-type PFFs were injected into egg cells to test whether the reconstructed embryos could develop normally. The development rates of the two types of blastocysts were similar (Figure 2B). A total of 450 recombinant embryos were transplanted into the uteruses of 5 surrogate sows and were detected by ultrasound after 28 days. Three surrogate sows were pregnant, and 10 newborn piglets were obtained after 118 days (Figure 2C). Specific primers (1F/1R) for PCR and Sanger sequencing revealed that 8 piglets were positive for the transgenes *IGF-1* and *Fat-1*, which were inserted at the p*Rosa26* locus (Figure 2D). RT-PCR confirmed that *IGF-1* and *Fat-1* can be effectively expressed in these positive clones. (Figure 2E).

### 3.3. Analysis of the IGF-1 Protein and Fatty Acids in the IGF-1 and Fat-1 Knock-in Pigs

**Figure 3:**
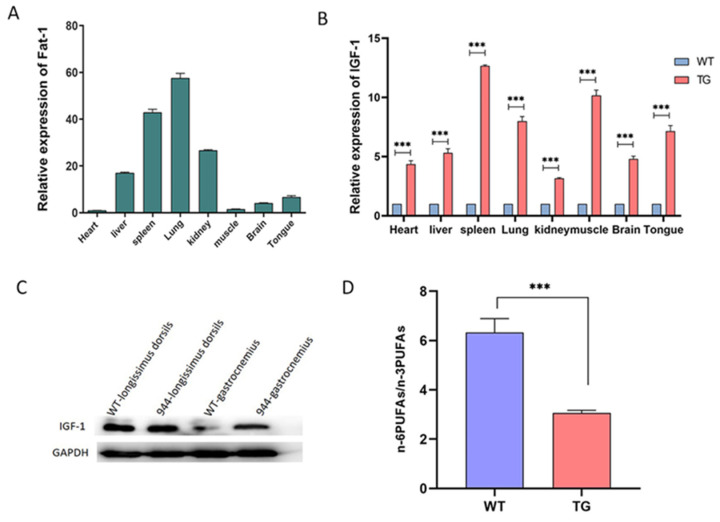
Expression analysis of the *Fat-1* and *IGF-1* genes. (**A**) Analyze the transcriptional expression of *Fat-1* in different tissues by real-time PCR. The values are denoted as the mean ± SEM, *n* = 3, *** *p* < 0.001. (**B**) Analyze the transcriptional expression of *IGF-1* in different tissues by real-time PCR.TG: transgenic pigs. WT: wild-type pigs. (**C**) Protein expression of *IGF-1* in muscle tissue as determined by Western blot. (**D**) The n-6 PUFAs/n-3 PUFAs ratio in *Fat-1* and *IGF-1* knock-in pigs and WT. TG: transgenic pigs. WT: wild-type pigs.

To identify the expression of *Fat-1* and *IGF-1* at the transcriptional level, we extracted total RNA from liver, kidney, spleen, heart, lung, and muscle tissues of positive transgenic pigs. Real-time fluorescence quantitative PCR results showed that the *Fat-1* and *IGF-1* genes were normally transcribed, and the relative expression of *Fat-1* was highest in the lung and lowest in the heart (Figure 3A). Compared with wild-type pigs of the same age, the expression of *IGF-1* was highest in the spleen of the transgenic pigs and lowest in the kidney (Figure 3B).

To further determine the protein expression of the *IGF-1* gene, total protein was extracted from the muscles of transgenic pigs and detected by Western blot. *IGF-1* was expressed at a significantly higher level in the gastrocnemius muscle of transgenic pigs than in the wild-type pig muscle, but there was almost no difference in its expression in the longissimus dorsi muscle between the two groups (Figure 3C).

To evaluate whether the expression of the *Fat-1* gene in transgenic pigs could alter the content of n-3 PUFAs, we extracted the total fatty acids from the muscles of transgenic and wild-type pigs as previously reported [33,34] and analyzed their fatty acid contents by gas chromatography. As we expected, the levels of LA and AA in transgenic pigs were decreased compared with those in wild-type pigs. On the contrary, the n-3 PUFAs level of transgenic pigs was significantly higher than that of wild-type pigs so that n-6 PUFA/n-3 PUFAs decreased significantly from 6.982 to 3.122 (Figure 3D and Table 1). In summary, the *Fat-1* gene can be expressed by the endogenous promoter of p*Rosa26* and convert n-6 PUFAs into n-3 PUFAs to reduce the n-6 PUFAs/n-3 PUFAs ratio.

### 3.4. Off-Target Analysis

**Figure 4:**
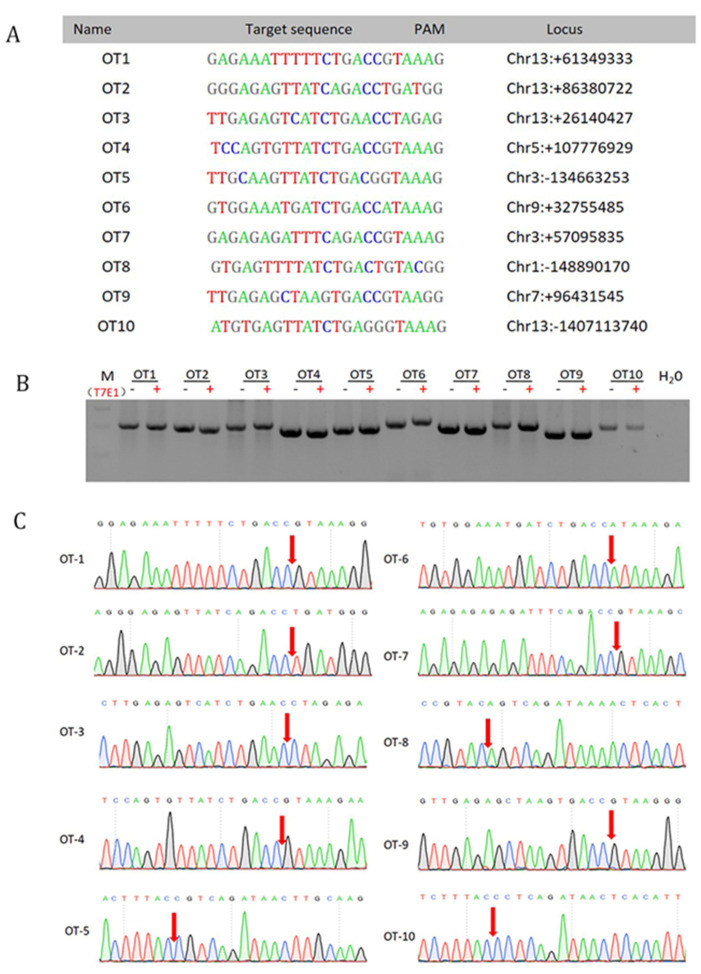
Analysis of off-target site. (**A**) The sequence and location of 10 potential off-target (OT) sites for sgRNA91/Cas9. (**B**) Cleavage analysis of 10 potential OT sites by T7E1. M: DNA 2000. (**C**) Sanger sequencing analysis of the PCR amplification products for 10 potential OT sites. The potential cleavage site of sgRNA91 has been marked with red arrow.

To ensure the effectiveness and safety of knock-in for these site-specific pigs, we selected total of 10 potential off-targets (OT) in the pig genome to detect the possibility of off-target (Figure 4A). The fragments around all 10 potential OT sites were analyzed by PCR amplification, and the PCR products were then analyzed for cleavage by the T7EI enzyme. No mutation was detected at any locus (Figure 4B). Similar to the results of T7EI digestion, Sanger sequencing showed no mutations at the 10 potential OT sites (Figure 4C). This result proves that CRISPR/Cas9 should be a reliable tool for foreign gene knock-in.

## 4. Discussion

As an important domestic animal, pigs play an important role in agricultural economy and human disease models. With the rapid development of CRISPR/Cas9 technology, research on genetically modified pigs has made great progress especially regarding production performance [1,3,6,35]. However, these studies almost exclusively integrated one foreign gene, and only one trait was improved for genetically modified pigs. This is far from sufficient for genetic breeding [36], xenotransplantation [37], and other transgenic research, which require the improvement of multiple simultaneously. Therefore, it is particularly important to integrate multiple foreign genes into the same site. However, there are many difficulties with this strategy such as a low integration efficiency, imbalanced expression between upstream foreign genes and downstream foreign genes, and low or even no expression of foreign genes. In our report, we used the self-cleaving 2A peptide and IRES technology, which allows multiple genes to be coordinately expressed from the same vector [28,38], to construct a system in which multiple genes are knocked in precisely at the same specific locus. In recent years, point-specific gene editing [39], selection of marker removal [40] and non-resistant gene screening have been studied by scholars to improve the safety of genetically modified animals. Some transgenic animals obtained through random integration may have unstable expression levels and phenotypes due to uncertain insertion sites of foreign genes [41,42]. In this report, the site specificity mediated by the CRISPR/Cas9 system allows foreign genes to be inserted into specific sites and stably expressed simultaneously [43,44], which avoids the ecological environmental risks due to the introduction of marker genes.

*IGF-1* is an important growth factor in mammals. Many studies have demonstrated that overexpression of *IGF-1* in skeletal muscle can promote muscle fiber hypertrophy and muscle production. Gao used α-actin protein regulators to drive the expression of *IGF-1* in mouse myoblasts and obtained transgenic mice with muscle hypertrophy [45]. Zou used the skeletal muscle-specific myosin light chain enhancer to upregulate the expression of *IGF-1* specifically in skeletal muscle and obtained muscle hypertrophy mice [46]. However, transgenic pigs with *IGF-1* gene integration at a specific site have rarely been reported. There are many reports on *Fat-1* genetically modified pigs. Lai prepared *Fat-1* transgenic pigs and reduced the ratio of n-6 PUFA/n-3 PUFA from 8.52 to 1.69 [42], while Li reduced the ratio from 9.36 to 2.12 [35]. Similarly, Zhang reduced the ratio of n-6 PUFA/n-3 PUFA in *Fat-1* transgenic pig muscle tissue from 48.85 to 10.91 [47]. To our knowledge, all of these pigs were modified with only one single foreign gene.

In this report, we used the CRISPR/Cas9 system to accurately integrate two genes into the p*Rosa26* locus, and the *Fat-1* and *IGF-1* genes were coexpressed by the self-cleaving 2A peptide and IRES technology [27,28]. With SCNT, we obtained 2 heterozygous positive piglets in which the *Fat-1* and *IGF-1* genes were expressed in various tissues. Protein-level analysis of *IGF-1* transgenic pigs showed that compared with wild-type piglets, the expression of the *IGF-1* gene in the gastrocnemius muscle was obviously different, while its expression in the longissimus dorsi was not significantly different between the WT and the genetically modified pig group. According to our analysis of fatty acids in the muscle tissue of transgenic pigs, the proportion of LA was reduced by 1.76-fold in *Fat-1* pigs compared with wild-type pigs, while that of AA was reduced by 0.68-fold. On the contrary, compared with wild-type pigs, the *Fat-1* pigs have increased ALA, DHA and DPA by 2.44 times, 2.09 times and 1.78 times, respectively. Therefore, the ratio of n-6 PUFAs/n-3 PUFAs in *Fat-1* pigs was decreased from 6.982 to 3.122. The site-specifically inserted *Fat-1* gene was successfully transcribed and expressed by the p*Rosa26* promoter under the regulation of 2A and converted n-6 PUFAs into n-3 PUFAs. Similarly, *IGF-1* was transcribed and expressed by the p*Rosa26* promoter under the regulation of the IRES.

## 5. Conclusions

We successfully produced site-specific *Fat-1* and *IGF-1* genetically modified pigs, which exhibited significantly reduced ratios of n-6 PUFAs/n-3 PUFAs and significantly increased *IGF-1* expression in muscle tissue. We successfully integrated multiple genes into the pig genome at a specific locus and simultaneously improved multiple transgenic pig traits, which benefits the health of consumers. Furthermore, the preparation of trans-genic animals with multigene targeted integration in this study provides a reference and new technical support for accelerating the precise integration of multiple exogenous genes, thereby providing technical support for the development of new genetically modified pig breeds with multiple excellent economic traits.

## Figures and Tables

**Table 1:** Analysis of PUFAs in wild-type pigs and positive pigs (*IGF-1* and *Fat-1* knock-in*)*.

Fatty Acids	Positive Pigs	Wild-Type Pigs
n-3 PUFAs		
C22:5(DPA)	22.195 ± 0.064221 ***	12.496 ± 0.053226 ***
C22:6(DHA)	12.042 ± 0.041313 ***	5.750 ± 0.038649 ***
C20:5(EPA)	39.160 ± 0.48597 ***	23.523 ± 0.002471 ***
C18:3(ALA)	18.553 ± 0.09573 ***	7.612 ± 0.021941 ***
Total n-3	91.951 ± 0.079628 ***	49.381 ± 0.029869 ***
n-6 PUFAs		
C18:2(LA)	138.318 ± 0.034776 ***	243.525 ± 0.066764 ***
C20:4(AA)	148.747 ± 0.058109 ***	101.243 ± 0.094351 ***
Total n-6	287.065 ± 0.010417 ***	344.768 ± 0.080017 ***
n-6/n-3 ratio	3.122	6.982

The data of each fatty acid in the table represents the percentage of this fatty acid to the total fatty acid. The values are denoted as the mean ± SEM, *n* = 3, *** *p* < 0.001.

## Data Availability

The authors stated that all the data needed to support the conclusions presented in the article are fully reflected in the article. All reagents and sources have been specified.
